# Cast Or ePineural Suture for digital nerve injuries (COPS): study protocol for a randomized controlled trial

**DOI:** 10.1186/s13063-026-09956-1

**Published:** 2026-08-26

**Authors:** Carin Carlsson, Johanna von Kieseritzky, Marianne Arner, Cecilia Mellstrand Navarro

**Affiliations:** 1 Department of Clinical Science and Education, Karolinska Institutet, Södersjukhuset, Stockholm, Sweden; 2 Department of Hand Surgery, Södersjukhuset Hospital, Stockholm, Sweden; 3 Department of Clinical Sciences, Section for Orthopaedics, Danderyd Hospital, Stockholm, Sweden

**Keywords:** Digital nerve, Digital nerve injury, Digital nerve suture, Digital nerve neurorraphy, Digital nerve repair

## Abstract

**Background:**

Injuries to digital nerves in the hand are very common and result in sensory disturbances, which can have a major negative impact on hand function. The gold standard of treatment is end-to-end nerve repair with sutures, at the latest within 2 weeks from injury. Despite operative treatment of digital nerve injuries, the clinical results are often poor. Recent literature questions the evidence supporting surgical repair of digital nerve injuries in adults.

**Methods:**

In this randomized controlled trial, we will allocate patients with injury to a single digital nerve to either surgical nerve repair and cast, or immobilization only. The primary outcome is the return of sensation, measured as static two-point discrimination, at 12 months after injury. Secondary outcomes include complications, the Semmes–Weinstein monofilament test, the Mini Sollerman hand function test, and patient-reported outcome measures.

**Discussion:**

The lack of high-quality studies comparing surgical repair of digital nerve injuries to simple immobilization of the finger motivates the conduct of this randomized study.

**Trial registration:**

ClinicalTrials.gov. TRN: NCT05536609. Registered on 08/23/2022.

**Supplementary Information:**

The online version contains supplementary material available at 10.1186/s13063-026-09956-1.

## Introduction

### Background and rationale {6a}

The digital nerves in the palm and fingers are the most frequent locations for peripheral nerve injuries [1]. The incidence is approximately 6.2–8.3/100.000 person-years [2, 3]. Hand function is essential for almost every activity, and a nerve injury with sensory loss or painful hyperesthesia can have a major negative impact on a person’s life. The need for adaptation of daily activities and the inability to perform previous work tasks, resulting in sick leave, have consequences for both the individual and society [3].

Despite surgical repair of nerve injuries, the clinical results can be poor [4]. Limited recovery of sensory function, development of cold intolerance, and painful neuroma in the affected finger are some of the remaining problems involved. A 2019 systematic review on the surgical repair of digital nerve injuries concluded that there is weak evidence to support digital nerve repair in adults with isolated digital nerve injuries [4]. A retrospective cohort study comparing sutured to non-sutured digital nerves found no difference in sensory recovery in groups with injuries distal to the middle of the middle phalanx [5]. A recently published randomized controlled study compared suture repair of digital nerve injury to alignment of the nerve ends. Results do not support beneficial effect of suture repair [6]. No randomized controlled study comparing operative treatment with immobilization alone has yet been published.

## Objectives {7}

The primary objective of this study is to investigate whether the return of static two-point discrimination (S2PD) after non-operative treatment is inferior to surgical repair of isolated digital nerve injury at 1-year follow-up.

Secondary objectives include comparison between the two groups for differences in patient-reported outcomes and hand function, including range of motion, grip strength, and additional aspects of sensation. We will also investigate complications and sick leave.

## Trial design {8}

This is a single-center, unblinded, superiority, randomized controlled trial. The study includes patients with isolated digital nerve injuries, defined as a single digital nerve laceration without concomitant injury to flexor or extensor tendons, or to adjacent digital nerves. Patients will be randomized in a 1:1 ratio, without stratification, to either surgical nerve repair and cast, or immobilization only.

## Methods: participants, interventions and outcomes

### Study setting {9}

The Department of Hand Surgery at Södersjukhuset Stockholm, Sweden, covers all specialized hand surgery, including nerve repairs, in the capital of Sweden. The catchment area has a population of approximately 2.5 million.

### Eligibility criteria {10}

Patients presenting with a suspected digital nerve injury will be referred to and examined at the Department of Hand Surgery at Södersjukhuset by the on-call surgeon, according to standard procedure. The eligibility criteria for this study will be assessed during the examination.

### Inclusion criteria

- Sharp cut, isolated single digital nerve injury
- Level of injury at the proximal or middle phalanx
-  ≥20 years of age at the time of injury
- Treatment/surgery possible within 10 days from injury

### Exclusion criteria

- Suspected concomitant tendon injury or fracture.
- Injury to the digital nerves in the thumb.
- Ongoing wound infection.
- Chronic neurological conditions.
- Cognitive disabilities and dementia.
- Ongoing drug or alcohol abuse.
- Injury > 7 days at examination

### Who will take informed consent? {26a}

At the time of examination, participants will receive both written and oral information about the study presented by either the on-call surgeon or a nurse. If the patient agrees to participate in the study, written informed consent will be obtained prior to enrollment.

### Additional consent provisions for collection and use of participant data and biological specimens {26b}

This study will not involve the collection of biological specimens, nor will participant data from this study be used in any ancillary studies.

## Interventions

### Explanation for the choice of comparators {6b}

There are no published randomized studies comparing results after surgical nerve repair to non-operative treatment of digital nerve injuries. Nevertheless, in 2025, the gold standard for treating digital nerve injuries is surgical treatment. A review study from 2019 was not able to show superior results in favor of either treatment of digital nerve injuries [4]. A non-randomized comparative study from 2023 reported improved S2PD following nerve repair in injuries proximal to the middle level at the middle phalanx. However, it found no significant differences in outcomes for sharp/dull or warm/cold sensation discrimination, complication rates, or patient-reported satisfaction [5]. In a recently published randomized controlled study, participants were randomized to either perioperative nerve suture or nerve alignment. The trial was closed early owing to a slow recruitment rate and therefore did not reach the intended sample size. However, the available data did not find one treatment superior to the other [6].

In this study, patients are randomized to either surgical nerve repair and cast immobilization or to cast immobilization alone, as both approaches are currently considered possible treatment options.

### Intervention description {11a}

At first examination, eligibility criteria, demographics, and information regarding the injury and its extent (perceived gender, date of birth, smoking, dominant hand, injured hand, profession, date of injury, injury mechanism, injured finger, injured nerve (ulnar/radial), level of injury (proximal or middle phalanx)) will be registered. Clinical examination will include sensory evaluation with S2PD of both the injured and the contralateral finger, as well as measurement of the distance between the injury and the fingertip in millimeters. Participants will be randomized to either nerve repair and cast or cast immobilization alone.

### Intervention group

Nerve repair will be performed under local, regional, or general anesthesia. The nerve will be repaired under loupe magnification, with epineural sutures using 8.0–9.0 nylon. Postoperative cast immobilization will be maintained for 3 weeks. The level of experience of the treating surgeon may range from that of a resident to that of a senior specialist.

### Control group

Cast immobilization will be maintained for 3 weeks. The cast will be administered by a nurse on the day of allocation.

In both groups, immobilization will consist of a cast over the wrist, stabilizing the metacarpophalangeal (MCP) joints in flexion and the proximal interphalangeal (PIP) joints in extension. Fingertips are left exposed to allow for sensory relearning exercises. Adjacent uninjured fingers will be included in the cast depending on the injury site and surgeon’s preference.

The follow-up protocols will be identical for both groups. All participants will be referred to an occupational therapist specialized in nerve rehabilitation to initiate early sensory relearning within 0–4 days following inclusion in the study. At 3 weeks, the cast and sutures will be removed. At this time, the occupational therapist will evaluate finger movement, sensation, strength, and overall hand function using standardized assessments, including active range of motion (AROM), S2PD, Semmes–Weinstein monofilament test (SWM), temperature and sharp/dull discrimination, the Mini Sollerman test, grip strength using the Jamar dynamometer, and Key pinch strength using the Pinch gauge. Follow-up evaluations will be conducted at 3, 6, and 12 months after inclusion (Table 1).

**Table 1:** COPS trial participant timeline

	Study period							
	Enrolment	Allocation	Post allocation/surgery					Close out
**Timepoint**	*-t*_*1*_	0	0–4 days	3 weeks	3 months	6 months	12 months	> 12 months
**Enrolment**								
Eligibility screen	X							
Informed consent	X							
Inclusion protocol		X						
Randomization surgery + cast or cast only		X						
**Intervensions**								
Sensory education			X					
Removal of cast and sutures				X				
**Assessments**								
Distance Injury—fingertip (mm)		X						
S2PD (mm)		X		X	X	X	X	
AROM (degrees)				X	X	X	X	
SWM (filament)				X	X	X	X	
Temperature (cold/warmth)				X	X	X	X	
Sharp/Dull				X	X	X	X	
Mini Sollerman test (points)				X	X	X	X	
Grip strength (kg) + VAS (0–10)				X	X	X	X	
Key pinch (kg) + VAS (0–10)				X	X	X	X	
**PROMs**								
Quick DASH		X			X	X	X	
HQ-8 before op		X						
HQ-8					X	X	X	
HADS		X						
PCS		X						
DN4				X	X	X	X	
**Other parameters**								
Demographics^a^	X							
Signs of infection (Yes or No)	X		X	X	X	X	X	
Sick leave (days)							X	

^a^Age, gender, smoking, handedness, occupation, comorbidities, injury mechanism, level of injury, time from injury to operation/immobilization

Participants will also receive general information about nerve injuries and rehabilitation, such as guidance on managing cold sensitivity and exercises to prevent finger stiffness. Patient-reported outcome measures (PROMs) focusing on hand function, pain, and psychological aspects will be collected (Table 1).

### Criteria for discontinuing or modifying allocated interventions {11b}

All participants are free to withdraw from study participation at any time. If any complications such as stiffness, infection, or pain should occur, they will be treated according to local routines. All results will be presented according to the intention-to-treat principle.

### Strategies to improve adherence to interventions {11c}

To improve adherence to the study protocol, the participants will be scheduled for all visits pertinent to the study follow-up. Questionnaires will be filled out during follow-up visits. If there are any questions, occupational therapists or other health care personnel will be available to assist with instructions. Participants who fail to attend follow-up visits will be contacted by phone or mail.

### Relevant concomitant care permitted or prohibited during the trial {11d}

No data or biological specimens from this study will be used in ancillary studies.

### Provisions for post-trial care {30}

Participants will not receive any compensation for their involvement in the study. The patients are covered by the Swedish patient injury insurance, Landstingets Ömsesidiga Försäkringsbolag (LÖF).

### Outcomes {12}

#### Primary outcome

Primary outcome measures will be collected at 12 months after injury.

### Static Two-Point Discrimination test (S2PD)

The primary outcome of this study is the return of sensation 12 months after nerve injury, assessed using the S2PD, measured in millimeters. S2PD is the most widely used test to evaluate sensory function in the fingers [7]. The S2PD test evaluates tactile spatial acuity i.e. the ability to distinguish between two separate points of contact on the skin. The S2PD test, initially described by Weber in 1834, and later modified by Moberg [7] is performed using the Mackinnon-Dellon Disk-Criminator™ by applying two pins longitudinally over the radial and ulnar sides of the fingertip, with distances ranging from 0 to 25 mm. The minimum distance at which an individual can accurately distinguish between one or two points is recorded as the S2PD value. The test will be conducted in a standardized manner, ensuring that the pins are applied with just enough pressure to cause skin blanching [8], as excessive force may stimulate other receptors rather than mechanoreceptors responsible for pressure sensation [9, 10]. The injured finger is examined, as well as the corresponding finger on the uninjured hand for comparison. A correct response in seven out of ten random applications is required for a valid measurement.

Classification of sensory recovery according to the Mackinnon-Dellon scale [11], modified from the Highet classification, will be used. Sensation is classified as: Poor = >15 mm, Good = 7–15 mm, Excellent = 2–6 mm. The test and its validity have been questioned [12, 13].

### Secondary outcomes

Secondary outcomes will be collected at inclusion, 3 weeks, 3, 6 and 12 months after injury.

### Active range of motion (AROM)

The range of motion of finger joints will be measured as degrees of motion in MCP, PIP, and distal interphalangeal (DIP) joints. A finger Goniometer will be used for measurements [14, 15] and the degrees of motion of the three finger joints will be summed. The injured finger and the corresponding finger on the uninjured hand will be measured. The measurement technique will adhere to the Swedish hand surgery quality registry (HAKIR) measurement manual [16]. Finger stiffness is a common complication of any finger injury.

### Semmes–Weinstein Monofilament test, pocket version (SWM)

This test assesses cutaneous sensory perception threshold and is widely used to evaluate protective sensation recovery after nerve injury1 [17]. Two versions of the test exist: the original version, which comprises 20 monofilaments of varying thicknesses, and a compact pocket version that uses five monofilaments. We will use the pocket version, and all data are presented according to the five monofilament categories. The monofilaments are applied perpendicularly to the hand and fingers (digit I, II, V, and the hypothenar region). Pressure is applied until the filament bends, indicating sufficient force [7, 8]. The injured finger and the corresponding finger on the uninjured hand will be examined.

### Temperature sensation

Assessment of temperature sensation as a binary outcome (cold/warmth) will be used to evaluate regeneration of nerve function [18]. The ability to discriminate between warm and cold stimuli is tested by gently applying a cold or heated aluminum spatula to the skin, distal to the injury. The spatulas have been heated or cooled using water of different temperatures. Sensation is often compromised after a nerve injury.

### Sharp/dull

The ability to distinguish between sharp and dull is often compromised after a nerve injury. This test is used to evaluate the return of sensibility.

Sharpness is tested with a safety pin lightly pressed against the fingertip, and dullness is tested with a cotton swab gently rubbed over the fingertip, distal to the site of injury [19]. A dull or sharp sensation is registered as a binary outcome.

### Mini Sollerman test

The Mini Sollerman test assesses hand function and dexterity and is conducted on both hands [20, 21]. It includes activities that incorporate various hand grips and hand function; picking up coins, handling screw-nuts, and buttoning. Tasks within the test are assigned scores ranging from 0 to 4. The total score is then divided by 12, with higher scores indicating better hand function. The Mini Sollerman test is a widely used instrument to assess hand function and dexterity. In patients with peripheral nerve injury (median and ulnar nerve), the Mini Sollerman test has been shown to be valid [22]. The test has not been evaluated for use in outcome measurement after digital nerve injuries. The HAKIR measurement manual for nerve injuries will be used [8].

### Grip strength

Grip strength is assessed using a Jamar dynamometer® [23]. The mean of three measurements will be presented in kilograms for both hands and as a percentage of the uninjured hand. Grip strength is often negatively affected by finger injuries and is commonly measured using this instrument. Jamar® has been shown to be reliable [15] and valid [23]. The HAKIR measurements manual will be adhered to [16].

### Key pinch

Key pinch, thumb tip pressed against the middle phalanx of the index finger, is used to measure strength and assessed by a pinch gauge [16, 23]. It will be recorded for both hands, and the mean of three measurements will be presented in kilograms, and as a percentage of the uninjured hand. Key pinch is often negatively affected by finger injuries and is commonly measured using this instrument. This test has also been shown to be reliable [23].

### Visual Analogue Scale (VAS)

During Jamar and pinch gauge assessments, the resulting intensity of pain will be rated by the patient on a straight line marked from 0 to 10 [24]. Where 0 is “no pain” and 10 is “worst possible pain” [24]. Rates from both hands will be recorded. VAS is a common self-assessment tool used to measure perceived pain.

### Patient-reported outcome measures (PROMs)

#### Short version of the Disabilities of the Arm, Shoulder and Hand questionnaire (Quick-DASH)

The Quick-DASH questionnaire is a region-specific validated measure of the perceived disability in the upper extremities, focusing on physical activity, pain, and symptoms [25]. It consists of 11 questions, and was developed from the original DASH questionnaire, which consisted of 30 questions [25, 26]. Each question is given a score between 1 and 5. To calculate the final score, the results of all questions are summed up, divided by the number of questions answered, subtracted by 1, and multiplied by 25. The total score ranges from 0 to 100, where 100 indicates maximal perceived disability. Not more than one missing item is allowed. The minimal clinically important difference has been reported to be a difference of 15–20 [27, 28].

### The HAKIR-8 questionnaire (HQ-8)

The HQ-8 questionnaire was constructed for evaluating patient-reported outcomes after hand surgery [29] and has been used since 2010 [17]. The HQ-8 includes seven questions concerning perceived symptoms in the affected or injured hand (pain on load, pain in motion without load, pain at rest, stiffness, weakness, numbness, and cold sensitivity) and one item about the ability to perform daily activities. Items are scored on a numeric 11-point box scale (NRS11) with numerical descriptors (0, 10, 20,…0.100) indicating no or maximal symptoms. The preoperative and the postoperative questionnaires are identical, with the exception that a question about the satisfaction with the result, scored 0–100, is added postoperatively. The questionnaire was adapted for the study by adding a “Not Relevant” option to the question regarding the “operated hand,” since not all patients will be operated. Its validity has been studied and is shown to be useful as a complement to Quick-DASH after hand surgery [29].

### Hospital Anxiety and Depression Scale (HADS)

HADS aims to measure symptoms of anxiety and depression [30]. It is a self-assessment questionnaire consisting of 14 items, seven about anxiety and seven about depression. Each question is scored 0–3. If the total score exceeds 10 in either anxiety or depression items, an anxiety or depression disorder is likely to be present, and it can increase the experience of post-surgical pain [30].

### Pain Catastrophizing Scale (PCS)

The psychological ability to cope with pain differs from individual to individual. Pain catastrophizing might be a personality risk factor for post-surgical pain [31]. The PCS was developed with the aim of identifying individuals who were more susceptible to experiencing pain than others [32]. The self-reported measure includes 13 items that assess feelings of helplessness, rumination, and magnification experienced in relation to pain. Responses are scored on a scale from 0 to 4, reflecting the frequency of these experiences. The maximum score is 52. A total score > 30 represents a clinically significant level of pain catastrophizing. High scores, > 30, might be a risk factor for post-surgical pain. Elevated levels of helplessness, rumination, and magnification, as identified by the PCS, are also common in individuals with anxiety and depression, indicating a potential overlap in psychological factors contributing to pain perception [33]. The scale was validated in Swedish in 2019 [34].

### Douleur Neuropathique en 4 questions (DN4)

This questionnaire is used as a screening instrument for neuropathic pain. The DN4 has been reported to be superior in sensitivity and specificity in identifying neuropathic pain [35]. The questionnaire is composed of ten items, with seven questions designed for patient response and three for the examiner to complete. The maximum attainable score is ten, and a score of four or higher suggests the probable presence of neuropathic pain. Neuropathic pain is a dreaded complication of nerve injury and will be reported as the result of the DN4 [35]. DN4 was translated into Swedish in 2011 and had the highest accuracy compared to three other tests when comparing neuropathic pain in patients with spinal cord injury [36].

### Complications

#### Neuropathic pain/neuroma

Suturing a digital nerve is considered to reduce the risk of neuroma development. Patients with a DN4 score of ≥4 are considered to exhibit signs of neuropathic pain and will undergo further examination.

### Cold intolerance

In the DN4 questionnaire, the first question (1:2) addresses cold and pain, and so does question number 7 in the HQ-8 questionnaire. The results from these questions will be analyzed separately from the rest of the questionnaires in order to assess cold intolerance. Cold intolerance is the most common complication after digital nerve injury [37].

### Wound infection

Symptoms consistent with infection (redness, swelling, pain, pus) will be recorded as a binary outcome, “yes” or “no”. Symptoms will be treated according to local routines.

### Other outcomes

#### Demographics

Data, including age (years), perceived gender (male/female), smoking (yes/no), handedness (right/left/ambidexter), occupation, comorbidities, injury mechanism, level of injury (middle or proximal phalanx), and time from injury to operation/immobilization (days), will be collected at inclusion.

### Sick leave

Sick leave will be documented and presented as the number of work absence days from inclusion to 1-year follow-up. A prolonged absence from work could potentially be caused by complications such as neuropathic pain. This might serve as an additional indicator of possible poor outcomes.

### Participant timeline {13}

#### Sample size {14}

This study was designed as a prospective superiority trial, aiming to determine whether one treatment strategy resulted in a superior sensory recovery outcome compared to the other. The primary endpoint was the proportion of participants achieving recovery of sensibility, defined as S2PD ≤ 10 mm, measured at the 1-year follow-up.

A power calculation was performed based on the assumption of a 20% clinically meaningful difference between groups. Using a two-sided significance level of *p* < 0.05 and a statistical power of 80%, the required sample size was calculated to be 150 patients. This calculation was based on an expected outcome proportion derived from a previously published study [38]. To account for an anticipated dropout rate of 10%, the total sample size was adjusted to 166 participants.

### Recruitment {15}

Inclusion started on the 29th of August 2022. The closing date is anticipated to 31 st of December 2030. All patients seeking care in the Södersjukhuset hospital due to a suspected digital nerve injury are screened for inclusion and exclusion criteria, and all eligible patients are invited to participate. All eligible individuals who decline participation will have their reason for non-participation documented. Additionally, for participants who withdraw from the study or follow-up, their reason for withdrawal will be recorded, if possible.

## Assignment of interventions: allocation

### Sequence generation {16a}

The allocation sequence was generated using block randomization and was prepared before the start of inclusion. Each block contains 10 tickets labeled “cast” and 10 tickets labeled “operation,” except for the last block, which consists of 6 tickets (three of each), as the total number of included patients was set to 166. Each ticket in one block was then randomly put in an opaque sealed envelope labeled 1–20, 21–40, etc.

### Concealment mechanism {16b}

The allocation is concealed in opaque, sealed envelopes.

### Implementation {16c}

The doctor examining the patient enrolls the participant. Together with the research nurse, an envelope (in consecutive order) is drawn. Either the doctor or the nurse informs the participant of the allocation.

## Assignment of interventions: blinding

### Who will be blinded {17a}

Interventions are not blinded, neither to healthcare personnel nor the patient.

### Procedure for unblinding if needed {17b}

Not applicable, as the interventions are not blinded.

## Data collection and management

### Plans for assessment and collection of outcomes {18a}

Data will be collected at inclusion and during follow-up visits at 0–4 days, 3 weeks, 3, 6, and 12 months from injury (Table 1). At the time of inclusion, written informed consent will be obtained, and the inclusion protocol, including inclusion and exclusion criteria as well as demographic data (age, gender, smoking habits, handedness, occupation, comorbidities, injury mechanism, level of injury, injured hand, injured finger, injured nerve, distance from injury to fingertip, and time from injury to treatment), will be completed. After consenting to participate, randomization will be carried out, and PROMs (Quick-DASH, HADS, HQ-8 (before operation), PCS) will be filled out. Sensory relearning is offered 0–4 days after injury. At 3 weeks, the cast and sutures will be removed by a nurse before seeing the occupational therapist. At follow-up visits, the investigating occupational therapist uses the follow-up study protocol for assessments (S2PD, AROM, SWM, Mini Sollerman test, Temperature sensation, Sharp/Dull, Grip strength + VAS, Pinch grip + VAS) and PROMs (Quick-DSAH, HQ-8 after operation/treatment, DN4), to make sure that all measurements and forms are completed.

### Plans to promote participant retention and complete follow-up {18b}

Participants who fail to complete questionnaires or to attend follow-up visits will be contacted by phone and/or mail by study group members.

### Data management {19}

Protocols and PROMs are completed on paper and stored in the participant’s file, which is kept in a locked archive space only accessible to the study group.

Data is entered from paper protocols into a computerized statistical program. Data accuracy will be verified through continuous review to check for input errors.

### Confidentiality {27}

Information about participants may be shared within the research team, research nurse, and occupational therapists from the date of inclusion through to 1-year follow-up. Follow-up protocols and PROMs on paper are stored in a locked archive space at Södersjukhuset hospital. At the end of the follow-up period, data is transferred from paper protocols into a computerized statistical program in pseudonymized form. The file is stored in a password-protected domain. The key linking the sequential number to the personal identification number is stored for 10 years in a locked archive space at Södersjukhuset.

### Plans for collection, laboratory evaluation and storage of biological specimens for genetic or molecular analysis in this trial/future use {33}

Not applicable, as no laboratory or biological specimens will be collected in this study.

## Statistical methods

### Statistical methods for primary and secondary outcomes {20a}

Primary outcome is a dichotomous outcome that will be presented as proportions of patients obtaining return of 2PD < 10 mm 1 year after injury, presented with 95% confidence interval, and proportions between groups will be compared with Chi^2^ analysis.

All numerical values will be tested for normality by Shapiro–Wilk’s test.

We assume normality/parametric data for age, AROM, and grip strength. Results are presented as numerical values on an interval scale. Central tendencies of data will be described as means with their corresponding standard deviation, and comparisons between groups will be performed with Student’s *t*-test or repeated measures ANOVA.

Categorical data will be presented as proportions and compared by Chi^2^ analysis, unless Fisher’s exact test is demanded due to expected small numbers. Ordinal data will be presented as medians and interquartile range (IQR), and Mann–Whitney *U*-tests or Wilcoxon’s test used for comparisons between groups.

We assume a skewed distribution for time to injury, sick leave, and PROMs, which will be presented as medians with IQRs and compared by Mann–Whitney *U*-tests or Wilcoxon’s test.

### Interim analyses {21b}

An interim analysis of adverse side effects will be conducted during the study. Once 42 patients, representing half of the planned study population, in each group, have completed the 6-month follow-up, the research team will assess the incidence of neuropathic pain. This assessment will be conducted using the DN4 questionnaire. A potential neuroma is considered present if a patient scores ≥4 on the DN4. If *a* ≥ 400% difference is observed in the proportion of patients scoring ≥4 on the DN4 questionnaire between the treatment groups, the study will be terminated due to an unacceptably high incidence of neuropathic side effects. Assessments of function, pain, and unwanted side effects will be conducted during follow-up visits, with regular evaluations of study participants held in collaboration with occupational therapists and researchers. If a patient scores ≥4 on DN4 and does not respond to conservative treatment for neuropathic pain (desensitization), he/she will be assessed by a hand surgeon for further diagnostics.

### Methods for additional analyses (e.g. subgroup analyses) {20b}

The primary outcome will be analyzed with mixed models effects, taking possible confounders into account, such as age (years), gender (female/male), smoking (yes/no), handedness (left/right), injury mechanism, level of injury (proximal/middle phalanx), distance from injury to fingertip (mm), time from injury to operation/immobilization (days). No other additional or subgroup analyses are planned.

### Methods in analysis to handle protocol non-adherence and any statistical methods to handle missing data {20c}

The main outcome in the final analysis will be presented according to the intention to treat principle. No imputation will be performed. Complications will be handled with an as-treated analysis.

### Plans to give access to the full protocol, participant level-data and statistical code {31c}

This document provides the complete study protocol. Pseudonymized data will be accessible on a reasonable request after the study has been completed.

## Oversight and monitoring

### Composition of the coordinating centre and trial steering committee {5d}

The group responsible for the study reviews the accuracy of the study data on a monthly basis.

### Composition of the data monitoring committee, its role and reporting structure {21a}

This study does not have a data monitoring committee or a sponsor. It is a single-center, randomized controlled trial, and no data is stored outside the hospital. No external party has access to data.

### Adverse event reporting and harms {22}

Complications and reported adverse events will be reported. Differences in adverse events between groups will be investigated at the time of the interim analysis and at the completion of the study.

### Frequency and plans for auditing trial conduct {23}

No auditing trial conducts are planned for this study.

### Plans for communicating important protocol amendments to relevant parties (e.g. trial participants, ethical committees) {25}

An amendment to the ethical approval has been submitted, requesting an extension of the study’s end date due to a low inclusion rate. No other significant modifications have been made since the study was designed.

## Dissemination plans {31a}

Results will be presented in a peer-reviewed journal and at national and international hand surgery meetings. Study participants will be notified when the study is published, either by mail or email.

## Discussion

This randomized controlled trial addresses an important and clinically relevant uncertainty regarding the optimal management of digital nerve injuries by evaluating whether surgical treatment with epineural sutures provides superior outcomes compared with non-operative treatment in a plaster cast. In addressing this clinically relevant uncertainty, the trial limitations should be considered. Despite the randomized controlled design, potential sources of bias remain, including selection bias during recruitment and attrition bias due to loss to follow-up. The current gold standard of treatment for digital nerve injuries is surgical treatment with neuroraphy. Patients willing to undergo randomization between treatment strategies that deviate from established standard-of-care pathways may not be fully representative of the broader patient population, potentially limiting the external validity of the trial. The study is not blinded. Therefore, the risk of outcome assessment bias will also be present. Participants who know they received the “recommended treatment” may be more likely to report improvement than those who did not. The occupational therapists performing the follow up measures, may unconsciously rate outcomes differently depending on the participant’s treatment group. These limitations should be considered alongside several important strengths. The randomized controlled design provides a robust framework for evaluating the comparative effectiveness of the interventions. The inclusion and exclusion criteria were carefully selected to define a clinically relevant study population, while the primary outcome was defined a priori and selected based on its widespread use thus facilitating comparisons with previous studies. Furthermore, the sample size calculation was based on prespecified and clinically justified assumptions to ensure adequate statistical power. Finally, prospective publication of this study protocol enhances transparency and methodological rigor by documenting the prespecified study design, outcomes, and analytical approach, thereby reducing the risk of selective outcome reporting and facilitating assessment of adherence to the original study plan. 

## Trial status

This is the first version of the study protocol. The protocol is available at ClinicalTrials.gov, reference number NCT:05536609. Recruitment started on the 29th of August 2022. Completion of inclusion is anticipated on the 31 st of December 2030.

## Supplementary Information

Additional file 1: Supplementary material.

## Data Availability

The research group will have access to the final trial dataset. Data sharing of pseudonymized variables will be provided from the corresponding author upon reasonable request, after the completion of the analysis and dissemination of the study.

## Abbreviations

AROM: Active range of motion
DIP: Distal interphalangeal
DN4: Douleur Neuropathique en 4 questions
HADS: Hospital Anxiety and Depression Scale
HAKIR: National quality register for hand surgery
HQ-8: HAKIR-8 questionnaire
IQR: Interquartile range
MCP: Metacarpophalangeal
PCS: Pain Catastrophizing Scale
PIP: Proximal interphalangeal
PROMs: Patient-reported outcome measures
Quick-DASH: Short version of the Disabilities of the Arm, Shoulder and Hand questionnaire
S2PD: Static two-point discrimination
SD: Standard deviation
SWM: Semmes–Weinstein monofilament test
VAS: Visual analogue scale
