# Supplementary material for: Cast Or ePineural Suture for digital nerve injuries (COPS): study protocol for a randomized controlled trial
Source: Trials. 2026 Aug 26;27:548. doi: 10.1186/s13063-026-09956-1 (PMC13508327; doi:10.1186/s13063-026-09956-1)
Supplement: Supplementary file 1 [file 13063_2026_9956_MOESM1_ESM.zip › Follow-up protocol COPS.docx]

| **Inclusion no.** |  |  |  |  | | | | | **Woman** | | | | |  | | **Man** |  |  |
| --- | --- | --- | --- | --- | --- | --- | --- | --- | --- | --- | --- | --- | --- | --- | --- | --- | --- | --- |
|  | | | | |  |  |  |  |  |  |  |  |  |  |  |  |  |  |
| **Handsurgery Clinic** | | | | |  | | | | | | | | | | | | |  |
| **Examination date** (yymmdd): | | | | |  | |  |  | |  |  |  |  | |  |  | |  |
| **Days after injury (0-4)** | | | | |  | |  |  | |  |  |  |  | |  |  | |  |
| **3 weeks** | | | | |  | |  |  | |  |  |  |  | |  |  | |  |
| **3 months** | | | | |  | |  |  | |  |  |  |  | |  |  | |  |
| **6 months** | | | | |  | |  |  | |  |  |  |  | |  |  | |  |
| **12 months** | | | | |  | |  |  | |  |  |  |  | |  |  | |  |
| **Examined finger/fingers:** | | | | |  | | | | | | | | | | | | |  |
| **Type of early sensory re-learning** | | | | |  | | | | | | | | | | | | |  |

| **SENSATION** | **Monofilament** | | | | | | | | | | | |
| --- | --- | --- | --- | --- | --- | --- | --- | --- | --- | --- | --- | --- |
|  | Contralateral hand, same finger | | Dig 1 | | Dig 2 | | Dig 3 | | Dig 4 | | Dig 5 | |
|  |  |  | r | u | r | u | r | u | r | u | r | u |
|  | SWM | |  |  |  |  |  |  |  |  |  |  |
|  |  | | | | | | | | | | | |
|  | Injured finger | | Dig 1 | | Dig 2 | | Dig 3 | | Dig 4 | | Dig 5 | |
|  |  |  | r | u | r | u | r | u | r | u | r | u |
|  | SWM | |  |  |  |  |  |  |  |  |  |  |
|  | | | | | | | | | | | | |
|  | **S2PD** | | | | | | | | | | | |
|  | Contralateral hand, same finger | | Dig 1 | | Dig 2 | | Dig 3 | | Dig 4 | | Dig 5 | |
|  |  |  | r | u | r | u | r | u | r | u | r | u |
|  | S2PD | |  |  |  |  |  |  |  |  |  |  |
|  |  | | | | | | | | | | | |
|  | Injured finger | | Dig 1 | | Dig 2 | | Dig 3 | | Dig 4 | | Dig 5 | |
|  |  |  | r | u | r | u | r | u | r | u | r | u |
|  | S2PD | |  |  |  |  |  |  |  |  |  |  |
|  | | | | | | | | | | | | |
| **TEMP** | Warmth | Yes | No | | | | | | | | | |
|  | Cold | Yes | No | | | | | | | | | |
|  |  | | | | | | | | | | | |
|  | Sharp | Yes | No | | | | | | | | | |
|  | Dull | Yes | No | | | | | | | | | |
|  | | | | | | | | | | | | |
| **AROM** | **Injured finger** | | Dig 1 | | Dig 2 | | Dig 3 | | Dig 4 | | Dig 5 | |
|  | **Extension/Flexion** | | | | | | | | | | | |
|  | MCP | | **/** | | **/** | | **/** | | **/** | | **/** | |
|  | PIP | | **/** | | **/** | | **/** | | **/** | | **/** | |
|  | DIP | | **/** | | **/** | | **/** | | **/** | | **/** | |
|  |  | | | | | | | | | | | |
|  | **Contralateral finger** | | Dig 1 | | Dig 2 | | Dig 3 | | Dig 4 | | Dig 5 | |
|  | **Extension/Flexion** | | | | | | | | | | | |
|  | MCP | | **/** | | **/** | | **/** | | **/** | | **/** | |
|  | PIP | | **/** | | **/** | | **/** | | **/** | | **/** | |
|  | DIP | | **/** | | **/** | | **/** | | **/** | | **/** | |
|  | | | | | | | | | | | | |

| **STRENGTH** | **Grip strength:** | | | | (JAMAR in kg, mean value of 3 measures) | | | | | | | | | | | | | | | | | | | | | | | | |
| --- | --- | --- | --- | --- | --- | --- | --- | --- | --- | --- | --- | --- | --- | --- | --- | --- | --- | --- | --- | --- | --- | --- | --- | --- | --- | --- | --- | --- | --- |
|  | Transversal  grip | | | | Right | | | | | | | | | | | | Left | | | | | | | | | |  | | |
|  |  |  |  |  |  | | |  | | |  | |  | | | |  | |  | |  | | |  | | |  |  |  |
|  | VAS at load (0–10): | | | | | | | | | | | | | | | | | | | | | | | | | | | | |
|  |  | | | | | | | | | | | | | | | | | | | | | | | | | | | | |
|  | **Pinch grip, I, II** | | | | (Pinch in Kg, mean value of 3 measures) | | | | | | | | | | | | | | | | | | | | | | | | |
|  |  | | | | Right | | | | | | | | | | | Left | | | | | | | | | |  | | | |
|  |  |  |  |  |  | | |  | |  | | | |  | |  | |  | |  | | |  | | |  |  |  |  |
|  | VAS at load (0–10): | | | | | | | | | | | | | | | | | | | | | | | | | | | | |
|  | | | | |  | | | | | | |  | | | | | |  | | | | | | |  | | | |  |
| **HAND FUNCTION** | | | | |  | | | | | | |  | | | | | |  | | | | | | |  | | | |  |
|  | Sollerman test task no. 4,8,10 (max 12 scores) | | | | | | | | | | | | | | | | | | | | | | | |  | | | |  |
|  |  | | |  | | | | | | | | Right | | | | | | Left | | | | | | |  | | | |  |
|  | 4. pick up coins from a purse: | | | | | | | | | | |  | | | | | |  | | | | | | |  | | | |  |
|  |  | | |  | | | | | | | |  | | | | | |  | | | | | | |  | | | |  |
|  | 8. Put nuts on bolts: | | | | | | | | | | |  | | | | | |  | | | | | | |  | | | |  |
|  |  | | |  | | | | | | | |  | | | | | |  | | | | | | |  | | | |  |
|  | 10 Button four buttons: | | | | | | | | | | |  | | | | | |  | | | | | | |  | | | |  |
|  |  | | | | | | | | | | |  | | | | | |  | | | | | | |  | | | |  |
|  | Sum scores | | | | | | | | | | |  | | | | | |  | | | | | | |  | | | |  |
|  |  | | |  | | | | | | | |  | | | | | |  | | | | | | |  | | | |  |
| **PROMs** |  | | |  | | | | | | | |  | | | | | |  | | | | | | |  | | | |  |
|  |  |  | | | | |  | |  | | | | | |  | | | | | | |  | | | | | |  | |
|  | **QuickDash** |  |  | | |  | |  | | | | | | |  | | | | | | |  | | | | | |  | |
|  | **HQ8 after op** |  |  | | |  | |  | | | | | | |  | | | | | | |  | | | | | |  | |
|  | **DN4** |  |  | | |  | |  | | | | | | |  | | | | | | |  | | | | | |  | |
|  |  |  |  | | |  | |  | | | | | | |  | | | | | | |  | | | | | |  | |
|  | | | | | | | | | | | | | | | | | | | | | | | | | | | | | |
| **ADD. INFO** |  | | | | | | | | | | | | | | | | | | | | | | | | | | | | |
|  |  | | | | | | | | | | | | | | | | | | | | | | | | | | | | |
|  |  | | | | | | | | | | | | | | | | | | | | | | | | | | | | |
|  |  | | | | | | | | | | | | | | | | | | | | | | | | | | | | |
|  |  | | | | | | | | | | | | | | | | | | | | | | | | | | | | |
|  |  | | | | | | | | | | | | | | | | | | | | | | | | | | | | |
|  |  | | | | | | | | | | | | | | | | | | | | | | | | | | | | |
|  |  | | | | | | | | | | | | | | | | | | | | | | | | | | | | |
|  |  | | | | | | | | | | | | | | | | | | | | | | | | | | | | |
|  |  | | | | | | | | | | | | | | | | | | | | | | | | | | | | |
|  |  | | | | | | | | | | | | | | | | | | | | | | | | | | | | |
| **Occ. therapist:** |  | | | | | | | | | | | | | | | | | | | | | | | | | | | | |
