# Supplementary material for: Cast Or ePineural Suture for digital nerve injuries (COPS): study protocol for a randomized controlled trial
Source: Trials. 2026 Aug 26;27:548. doi: 10.1186/s13063-026-09956-1 (PMC13508327; doi:10.1186/s13063-026-09956-1)
Supplement: Supplementary file 1 [file 13063_2026_9956_MOESM1_ESM.zip › Inclusion protocol COPS.docx]

| **Inclusion criteria** | Sharp injury, isolated digital nerve injury, indication for operation | | | | | | | | | | | | **Yes** | | | | | **No** | |
| --- | --- | --- | --- | --- | --- | --- | --- | --- | --- | --- | --- | --- | --- | --- | --- | --- | --- | --- | --- |
|  | Level of injury on proximal or middle phalanx | | | | | | | | | | | | **Yes** | | | | | **No** | |
|  | Patient ≥ 20 years at injury | | | | | | | | | | | | **Yes** | | | | | **No** | |
|  | Operation is offered within 10 days from injury | | | | | | | | | | | | **Yes** | | | | | **No** | |
| **Exclusion criteria** | Concomittent tendon injury or fracture | | | | | | | | | | | | **Yes** | | | | | **No** | |
|  | Injury on thumb | | | | | | | | | | | | **Yes** | | | | | **No** | |
|  | Wound infection | | | | | | | | | | | | **Yes** | | | | | **No** | |
|  | Chronic neurologic disease, cognitive dysfunction, or dementia | | | | | | | | | | | | **Yes** | | | | | **No** | |
|  | Does not understand Swedish language | | | | | | | | | | | | **Yes** | | | | | **No** | |
|  | Alcohol or drug abuse | | | | | | | | | | | | **Yes** | | | | | **No** | |
|  | Injury> 7 days at injury | | | | | | | | | | | | **Yes** | | | | | **No** | |
| **Patient eligable for study** | | | | | | | | | | | | | **Yes** | | | | | **No** | |
|  |  | | |  | | | |  | | | | |  | | | | |  | |
| **Inclusion No.** |  | | |  | | | | **Date of inclusion:** | | | | |  | | | | |  | |
|  |  | | |  | | | |  | | | | |  | | | | |  | |
| **Gender** | male | | |  | | | | woman | | | | |  | | | | |  | |
|  |  | | |  | | | |  | | | | |  | | | | |  | |
| **Date of birth** year |  | | | month | | | |  | | | | | day | | | | |  | |
|  |  | | |  | | | |  | | | | |  | | | | |  | |
| **Smoking** | Never | | | | | Used to | | |  | | | Now and then | | | |  | | | Daily |
|  |  | | |  | | | |  | | | | |  | | | | |  | |
| **Handedness** | Left | | |  | | | | Right | | | | |  | | | | | Both | |
|  |  | | |  | | | |  | | | | |  | | | | |  | |
| **Injured hand** | Left | | |  | | | | Right | | | | |  | | | | |  | |
|  |  | | |  | | | |  | | | | |  | | | | |  | |
| **Occupation** | |  | | | | | | | | | | |  | | | | |  | |
|  |  | | |  | | | |  | | | | |  | | | | |  | |
| **Randomized for** | Operation + cast | | |  | | | | Cast | | | | | | | | | |  | |
|  |  | | |  | | | |  | | | | |  | | | | |  | |
| **Date of injury** | Year | |  | | | | Month | | |  | | | | Day | | |  | | |
|  |  | | |  | | | |  | | | | |  | | | | |  | |
| **Mechanism of injury** |  | | | | | | |  | | | | |  | | | | |  | |
|  |  | | |  | | | |  | | | | |  | | | | |  | |
| **Injured finger** | Index finger | | | | Long finger | | | | | | Ring finger | | | | Little finger | | | | |
|  |  | | |  | | | |  | | | | |  | | | | |  | |
| **Injury, ulnar or radial** | ulnar | | |  | | | | radial | | | | |  | | | | |  | |
|  |  | | |  | | | |  | | | | |  | | | | |  | |
| **S2PD injured nerve** | mm | | |  | | | | **S2PD same nerve contralateral hand** | | | | | | | | | | mm | |
|  |  | | |  | | | |  | | | | |  | | | | |  | |
| **Level of injury** | | | | | | | | Proximal  phalanx | | | | | Middle phalanx | | | | |  | |
|  |  | | |  | | | |  | | | | |  | | | | |  | |
| **Distance injury-fingertip** | | | | | | | | | | | | | mm | | | | |  | |
|  |  | | |  | | | |  | | | | |  | | | | |  | |
| **HADS, PCS, HQ-8 before op, Quick-DASH filled out** | | | | Yes | | | | **Written consent** | | | | | | | | | | Yes | |
|  |  | | |  | | | | **Surgeons signature** | | | | |  | | | | |  | |
