# Supplementary material for: Cast Or ePineural Suture for digital nerve injuries (COPS): study protocol for a randomized controlled trial
Source: Trials. 2026 Aug 26;27:548. doi: 10.1186/s13063-026-09956-1 (PMC13508327; doi:10.1186/s13063-026-09956-1)
Supplement: Supplementary file 1 [file 13063_2026_9956_MOESM1_ESM.zip › Study protocol COPS.docx]

| **Inclusion** | **Nurse**  - Study information to patient (written and oral)  - Fill out inclusion protocol (red parts)  - Tickets for randomization  **Surgeon**  - Fill out inclusion protocol (red parts)  - Randomization  - Plan for operative treatment or not  **Participant**  -Signe consent  -Quick-DASH  -HADS  -HQ-8 (before operation)  -PCS |
| --- | --- |
| **Day 0-4** | **Occupational therapist**  - Follow-up protocol  - Early sensory re-learning |
| **3 Weeks** | **Nurse**  - Remove cast and stitches  **Occupational therapist**  Follow-up protocol:  - Semmes-Weinstein monofilament test (SWM  - Statisk 2-punktsdiskrimination (S2PD)  - Temperature Warmth/Cold  - Sharp/Dull  - Active Range of Motion (AROM)  - Grip strength  - Pinch grip  - Mini Sollerman test  **Patient**  - Quick DASH  - HQ8 (after operation)  - Douleur Neuropathique 4 questions (DN4)* |
| **3, 6 and 12 months** | **Nurse**  - Remove cast and stitches  **Occupational therapist**  Follow-up protocol:  - Semmes-Weinstein monofilament test (SWM  - Statisk 2-punktsdiskrimination (S2PD)  - Temperature Warmth/Cold  - Sharp/Dull  - Active Range of Motion (AROM)  - Grip strength  - Pinch grip  - Mini Sollerman test  **Patient**  - Quick DASH  - HQ8 (after operation)  - Douleur Neuropathique 4 questions (DN4)* |

*Two questions are filled out by the occupational therapist
